# Whole exome sequencing and polygenic risk assessment for kidney functions and clinical management in both hospital-based cohort and population-based Asian cohorts

**DOI:** 10.1186/s12929-025-01168-0

**Published:** 2025-08-06

**Authors:** Min-Rou Lin, I-Wen Wu, Wan-Hsuan Chou, Yung-Feng Lin, Kuan-Yu Hung, Kaname Kojima, Kosuke Shido, Kengo Kinoshita, Wei-Chiao Chang, Mai-Szu Wu

**Affiliations:** 1 Department of Clinical Pharmacy, School of Pharmacy, Taipei Medical University, Taipei, Taiwan; 2 Division of Nephrology, Department of Internal Medicine, Taipei Medical University Hospital, Taipei, Taiwan; 3 Division of Nephrology, Department of Internal Medicine, School of Medicine, Taipei Medical University, Taipei, Taiwan; 4 Taipei Medical University-Research Center of Urology and Kidney (TMU-RCUK), Taipei Medical University, Taipei, Taiwan; 5 Department of Pharmacy, Taipei Medical University Hospital, Taipei Medical University, Taipei, Taiwan; 6 Taipei Cancer Center, Taipei Medical University Hospital, Taipei Medical University, Taipei, Taiwan; 7 Department of Medical Research, Taipei Medical University Hospital, Taipei, Taiwan; 8 Division of Nephrology, Department of Internal Medicine, Shuang Ho Hospital, Taipei Medical University, New Taipei City, Taiwan; 9 Tohoku Medical Megabank Organization, Tohoku University, Sendai, Japan; 10 Department of Dermatology, Graduate School of Medicine, Tohoku University, Sendai, Japan; 11 Graduate School of Information Sciences, Tohoku University, Sendai, Japan; 12 Advanced Research Center for Innovations in Next-Generation Medicine, Tohoku University, Sendai, Japan; 13 Institute of Development, Aging and Cancer, Tohoku University, Sendai, Japan; 14 Master Program in Clinical Genomics and Proteomics, School of Pharmacy, Taipei Medical University, Taipei, Taiwan; 15 Core Laboratory of Neoantigen Analysis for Personalized Cancer Vaccine, Office of R&D, Taipei Medical University, Taipei, Taiwan; 16 Integrative Research Center for Critical Care, Wan Fang Hospital, Taipei Medical University, Taipei, Taiwan; 17 Department of Pharmacy, Wan Fang Hospital, Taipei Medical University, Taipei, Taiwan; 18 School of Pharmacy, College of Pharmacy, Kaohsiung Medical University, Kaohsiung, Taiwan

**Keywords:** Chronic kidney disease, End-stage kidney disease, Rare variants, Whole-exome sequencing, Genetic susceptibility, Polygenic risk score, Disease risk stratification, Clinical management

## Abstract

**Background:**

Taiwan has the highest prevalence of chronic kidney disease (CKD) and end-stage kidney disease (ESKD) globally, making them major public health concerns with significant morbidity, mortality, and healthcare burden. While genetic risk factors for kidney disease have been identified in previous studies, the contribution of rare genetic variants remains unclear.

**Methods:**

This study utilized whole-exome sequencing (WES) to investigate the role of missense rare variants in CKD and ESKD susceptibility. Genomic data from 500 Taiwanese individuals at Taipei Medical University Hospital were included based on strict clinical diagnostic criteria, comprising 200 CKD cases, 200 ESKD cases, and 100 healthy controls. Independent validation was performed using ESKD Asian cohorts from the All of Us Research Program (AoU) (N = 222) and the Tohoku Medical Megabank Organization (ToMMo) (N = 140).

**Results:**

We identified rare pathogenic variants in known monogenic kidney disease genes, including *PKD1* and *COL4A4*, confirming their role in disease susceptibility. We replicated GWAS-reported genes such as *SPI1*, *RIN3*, *FTO*, *SIPA1L3*, and *EEF1E1*, highlighting their contribution through both common and rare variants. Beyond previously reported genes, we identified novel rare pathogenic variants in *PEX1*, *GANAB*, *DYNC2H1*, and *PROKR2*. Pathway enrichment analysis suggested that ciliopathies, inflammation, and metabolic dysfunction may contribute to kidney disease progression. Furthermore, the polygenic score (PGS) for ESKD demonstrated strong predictive utility for kidney function, with high genetic risk having a greater influence than comorbidities such as diabetes and overweight. The prediction power of ESKD PGS was further confirmed in the AoU Asian population.

**Conclusions:**

This study provides novel insights into the genetic architecture of CKD and ESKD in the Taiwanese population, utilizing a hospital-based cohort with strict clinical diagnostic criteria to ensure precise phenotype classification. We propose that individuals with high genetic risk may benefit from earlier interventions, while those with lower PGS may be better managed through lifestyle modifications targeting comorbidities. The findings highlight the importance of preventive strategies and precision medicine in kidney disease management.

**Supplementary Information:**

The online version contains supplementary material available at 10.1186/s12929-025-01168-0.

## Background

Taiwan has one of the highest rates of kidney disease, making it a significant public health issue due to its effects on morbidity, mortality, and healthcare expenses [1]. The prevalence of chronic kidney disease (CKD) in Taiwan is estimated to be 15.46% [2]. Patients with advanced stages of CKD, characterized by an estimated glomerular filtration rate (eGFR) lower than 15 mL/min/1.73 m^2^ with organ decompensation, are diagnosed with end-stage kidney disease (ESKD). ESKD patients require hemodialysis, peritoneal dialysis or renal transplantation, making Taiwan one of the countries with the highest dialysis treatment rates worldwide [3]. This emphasizes the urgent need to understand the genetic and environmental factors that contribute to CKD and ESKD.

Genetic variants influencing kidney function have been reported to affect a significant proportion of the Taiwanese population [4, 5]. The genetic causes of CKD can arise from either monogenic, polygenic, or cumulative interactions with environmental factors [6]. Notably, variants in the *APOL1* gene, found exclusively in Africans, confer a fourfold higher risk of developing CKD compared to Europeans and Americans [7]. Other monogenic causes include *COL4A3*, *COL4A4*, *COL4A5*, *HNF1B*, *MUC1*, *PKD1*, *PKD2* and *PKHD1* [8, 9]. Alternatively, genome-wide association studies (GWAS) have been instrumental in identifying common genetic loci associated with kidney function and CKD development [10]. However, these common variants explain only a fraction of the genetic basis of kidney diseases. A key limitation of GWAS findings lies in the trade-off between allele frequency and effect size: common variants with higher allele frequencies generally exhibit smaller effect sizes, whereas rare variants often have larger effects but are less frequent in the population. Consequently, rare variants may contribute more substantially to disease risk than previously estimated. Emerging evidence underscores the crucial role of rare variants in the genetic architecture of CKD. Investigating these variants can provide valuable insights into disease mechanisms that remain unclear when focusing solely on common genetic variants [11]. For example, Cameron-Christie et al. identified rare genetic variants associated with CKD subtypes in a predominantly Caucasian population [11]. Their findings not only replicate previously known CKD-associated genes but also uncover novel genes, such as *SCLT1*, *SLC17A1*, and *CPT2*. However, rare genetic germline mutations contributing to CKD and ESKD remain unclear in East Asian populations.

**Fig. 1:**
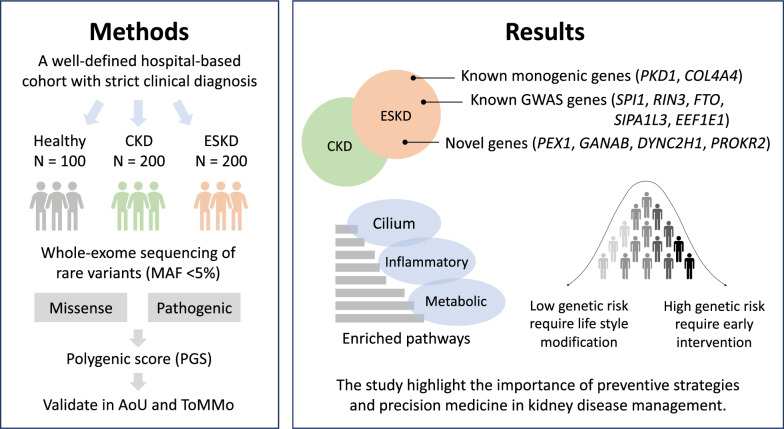
Overview of the Study Design. This study identifies missense rare variants associated with CKD and ESKD using WES data from a hospital-based cohort with 500 Taiwanese individuals. Pathway enrichment analysis was conducted to elucidate the biological functions of the identified genes. Functional annotation was performed to assess the pathogenicity and mutational consequences of the variants. Polygenic risk scoring was applied to estimate the cumulative genetic impact of these variants. Finally, we validate our findings in the Asian population cohort from AoU and ToMMo

This study aims to address this gap by investigating the role of rare variants in CKD and ESKD susceptibility. Utilizing exome-based rare-variant analyses, we analyzed 200 CKD cases, 200 ESKD cases, and 100 healthy individuals to gain a comprehensive understanding of the genetic basis of kidney diseases. Our study incorporated pathway enrichment analysis and functional annotation to identify key molecular mechanisms underlying CKD and ESKD. Additionally, polygenic scoring (PGS) was employed to assess the impact of identified variants on kidney-related traits. Validation was conducted using Asian cohorts from the All of Us Research Program (AoU) and the Tohoku Medical Megabank Organization (ToMMo) (Fig. 1). Our results have the potential to improve disease prediction and facilitate the development of targeted therapies for CKD and ESKD.

## Methods

### Study population in the TMU cohort

In the TMU cohort, all data were included from Taipei Medical University biobank, with the bio-specimen collected as appropriate and stored at − 80 °C until analysis. CKD and ESKD patients were age- and sex- matched with healthy individuals. The definition of CKD is based on diagnosis codes and laboratory data. Diagnosis codes include ICD-10: N18 (excluding N18.6, which indicates ESKD) or ICD-9: 585 (excluding 585.6, which indicates ESKD). Laboratory criteria include an eGFR < 60 mL/min/1.73 m^2^, a urine protein-creatinine ratio (uPCR) ≥ 150 mg/g, or a urine microalbumin-creatinine ratio (uACR) ≥ 30 mg/g. The definition of ESKD is based on diagnosis codes for ESKD, renal failure, renal dialysis, or kidney transplant. Diagnosis codes include ICD-10: N18.6, N19, Z99.2, Z94.0 or ICD-9: 585.6, 586, V45.1, V42.0. The definition of healthy individuals excludes those with CKD, ESKD, or any of the following conditions: ICD-10 codes for obesity (E66, O99.21, Z68.3, Z68.4), malignant neoplasms (C00–C97), diabetes (E08–E13), hypertension (I10–I15), coronary artery disease (I20–I25), heart failure (I50), or stroke (I60–I69). Similarly, exclusions by ICD-9 codes include obesity (278, 649.1, V85.3, V85.4), malignant neoplasms (140–208), diabetes (250), hypertension (401–405), coronary artery disease (410–414), heart failure (428), or stroke (430–438). The study was approved by the Institutional Review Board (IRB) of Taipei Medical University (N202404157). The written informed consents of the subjects were obtained upon recruitment to the TMU biobank.

### Whole exome sequencing and bioinformatic analysis

A total of 500 samples were sequenced by Illumina NovaSeq 6000 platform. Paired-end sequencing was conducted with sequencing depth of 100 × . The quality of the sequencing data was assessed by fastQC, fastQ screen, and multi-QC tools. Trimmomatic was used to exclude low-quality sequences. All sequences were aligned to the human reference genome GRCh38 and joint variant calling were conducted by GATK.

Biological information of each variant was obtained using ANNOVAR [12] and the Ensembl Variant Effect Predictor (VEP) tools [13]. Rare variants were defined as those with a minor allele frequency (MAF) of less than 0.05 in the Taiwan Biobank, East Asian population from the 1000 Genomes Project, and the East Asian population from the Genome Aggregation Database. The study focused on identifying genetic variants with high to moderate impact, selecting variants that fall into specific functional categories. These categories include: transcript ablation, splice acceptor variant, splice donor variant, stop gained, frameshift variant, stop lost, start lost, transcript amplification, feature elongation, feature truncation, inframe insertion, inframe deletion, missense variant, protein altering variant. Known kidney disease-related variants and genes were selected by accessing the GWAS catalog using the R package ‘gwasrapidd’ (access date on Feb 17, 2025). We particularly focused on variants that were reported to be associated with CKD (EFO_0003884), ESKD (EFO_0009909), and glomerular filtration rate (EFO_0005208).

### Pathogenicity annotation and pathway enrichment analysis

Pathogenicity was defined by four approaches: (1) ClinVar (v.20240611) with variants annotated as either conflicting classifications of pathogenicity, pathogenic, or likely pathogenic; (2) CADD Phred score > 20; (3) Polyphen-2 classified as damaging or possibly damaging; (4) SIFT classified as damaging. Pathway analysis was performed using over-representation analysis (ORA) with the ‘ClusterProfiler’ R package. We utilized the Gene Ontology biological process (GOBP) [14, 15], the Kyoto Encyclopedia of Genes and Genomes (KEGG) [16], WikiPathways [17], and Reactome [18] to identify enriched pathways. Gene Ontology (GO) is a framework that provides a structured and controlled vocabulary for describing the functions of genes. The Biological Process (BP) specifically focuses on events that lead to a specific biological outcome. KEGG provides curated sets of biological pathways representing the functional networks of gene products and their interactions. WikiPathways and reactome are open and collaborative platforms that allows researchers to update information of biological pathways. The Benjamini–Hochberg method was applied to adjust for multiple testing, with an adjusted p-value of < 0.05 considered statistically significant. The top five enriched pathways identified by each of the four tools were reported using their original annotations. Overlapping pathways across different databases were highlighted and discussed in the study.

### Statistical analyses and polygenic scoring (PGS)

To identify variants significantly associated with CKD or ESKD, we used fisher’s exact test to compare the number of carriers of each missense rare variant between healthy individuals and patients with CKD or ESKD. Calculations were performed in R software v4.2 to generate p-values and odds ratio. To quantify the cumulative effect of our findings, we established a PGS for CKD and ESKD based on the summary statistics of fisher’s exact test. Variants with p-value of < 0.05 were included in the calculation of PGS. The odds ratio of each variant was converted to beta [log(OR)] to serve as the effect size for calculating the polygenic score, allowing the contribution of each variant to be modeled additively. The comparison of eGFR across PGS groups was performed using Wilcoxon rank-sum tests.

### Validation cohorts from the all of us research program (AoU) and the Tohoku medical megabank organization (ToMMo)

The WES data from the AoU v8 cohort was utilized to validate the PGS for ESKD, focusing specifically on the Asian population. Ancestry was determined based on self-reported participant responses. To define the ESKD cohort, we included individuals diagnosed with ESKD (ICD-10: N18.6 and ICD-9: 585.6), those who had undergone kidney transplantation (ICD-10: Z94.0 and ICD-9: V42.0), or those receiving renal dialysis (ICD-10: Z99.2 and ICD-9: V45.1). For the healthy cohort, we applied the same exclusion criteria same as the TMU cohort and further exclude individuals with CKD (ICD-10: N18 and ICD-9: 585), ESKD (ICD-10: N18.6, Z94.0, Z99.2 and ICD-9: 585.6, V42.0, V45.1), hyperlipidemia (ICD-10: E78 and ICD-9: 272), hyperparathyroidism (ICD-10: E21 and ICD-9: 252), hematuria (ICD-10: R31 and ICD-9: 599.7), cardiac dysrhythmias (ICD-10: I49 and ICD-9: 427), peripheral vascular disease (ICD-10: I73 and ICD-9: 443.8), cystic kidney disease (ICD-10: Q61 and ICD-9: 753.1), or Alport syndrome (ICD-10: Q87.81). Controls were individually matched 1:1 to ESKD cases by sex and age. Only individuals with a recorded sex assigned at birth as male or female were included in the cohort. The informed consents of the AoU subjects were obtained in person or via electronic platform upon their enrollment into the AoU Research Program. Furthermore, whole-genome sequence (WGS) data from participants of the ToMMo cohort study were used. ESKD cases were identified based on questionnaire responses indicating dialysis and free-text reports referencing kidney transplantation or kidney failure. In addition, samples with an eGFR < 15 mL/min/1.73 m^2^ were included as cases. From the ToMMo control cohort, we applied the same exclusion criteria same as the TMU cohort and further exclude participants with HbA1c (as standardized by the National Glycohemoglobin Standardization Program, NGSP) ≥ 6.5 due to potential diabetes. Controls were then selected by sex and age matching, with a control-to-case ratio of 3:1. The written informed consents of the ToMMo subjects were obtained upon their enrollment into the biobank.

## Results

### Overview of study cohort and patient baseline characteristics

A total of 500 participants were included for this study, comprising 200 individuals diagnosed with CKD, 200 with ESKD, and 100 healthy controls (Table 1). The participants ranged in age from 21 to 88 years, with a mean age of 61.9 years. As expected, healthy individuals exhibited the highest median eGFR level (88.5 mL/min/1.73 m^2^), while CKD patients had a significantly lower median eGFR of 58.35 mL/min/1.73 m^2^, and ESKD patients had the lowest at 6.52 mL/min/1.73 m^2^. Comorbid conditions were prevalent among CKD and ESKD patients, which may contribute to clinical and genetic heterogeneity within the cohort. Hypertension was observed in 35% of CKD cases and 93% of ESKD cases. Diabetes was present in 23.5% of CKD cases and 63.5% of ESKD cases. Chronic glomerulonephritis was reported in 17% of CKD patients and 16.5% of ESKD patients. Autoimmune diseases, including Sjögren’s syndrome and systemic lupus erythematosus (SLE), were identified in 5 CKD patients and only 1 ESKD patient. In contrast, no comorbidities were observed in the healthy control group. WES analysis identified a total of 6,325,868 germline variants across all participants. After applying stringent filtering criteria, including the selection of rare missense variants as outlined in the Methods section, 37,263 variants remained for downstream analysis (Fig. 1). A variant-based association test was conducted in a cohort of 300 individuals each for CKD and ESKD. The analysis identified 459 rare variants significantly associated with CKD, including 448 missense variants, 9 stop-gained variants, 1 start-lost variant, and 1 splice donor variant (Supplementary Table 1). Similarly, 473 rare variants were significantly associated with ESKD, comprising 459 missense variants, 3 splice acceptor variants, 2 stop-gained variants, and 1 splice donor variant (Supplementary Table 2). Between CKD- and ESKD-associated variants, 136 overlapped, suggesting a substantial shared genetic background between the two diseases. All shared variants exhibited consistent effect directions across CKD and ESKD. Additionally, all CKD-associated variants were mapped to 435 genes, while ESKD-associated variants were identified in 436 genes.

**Table 1:** Baseline characteristics of subjects in the study

	All	CKD	ESKD	Healthy
Number	500	200	200	100
Age (years, mean ± SD)	61.93 ± 13.04	61.87 ± 13	61.74 ± 13.13	62.45 ± 13.07
Sex (male, %)	62.6%	62%	63%	63%
BMI (mean ± SD)	26.08 ± 9.74	25.47 ± 3.68	27.41 ± 13.92	23.52 ± 2.94
eGFR (mL/min/1.73 m^2^, median [q1, q3])	39.95 [7.76, 71.07]	58.35 [45.94, 75.17]	6.52 [4.79, 12.73]	88.50 [76.40, 100,79]
uPCR (mg/g, median [q1, q3])	244.69 [91.05, 1469.0]	151.14 [78.46, 557.43]	2295.11 [1121.81, 6247.04]	83.36 [69.67, 106.31]
uACR (mg/g, median [q1, q3])	161.71 [20.70, 1537,39]	41.50 [12.08, 249.01]	1750.62 [562.72, 3376.14]	4.36 [4.17, 7.81]
Overweight (BMI≧23, %)	56.2%	66%	59%	31%
Hypertension (%)	51.2%	35%	93%	0%
Diabetes (%)	34.8%	23.5%	63.5%	0%
Autoimmune disease (%)	1.2%	2.5%	0.5%	0%
Chronic glomerulonephritis (%)	14%	17%	16.5%	0%

Autoimmune disease refers to Sjögren’s syndrome or systemic lupus erythematosus (SLE)

BMI: body mass index; eGFR: estimated glomerular filtration rate; uPCR: urine protein-creatinine ratio; uACR: urine microalbumin-creatinine ratio

### Rare pathogenic variants and their association with CKD and ESKD

**Fig. 2:**
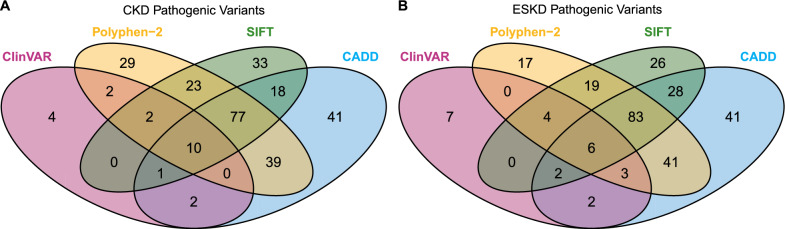
Pathogenic missense rare variants associated with CKD and ESKD. **A** Venn diagram of CKD-associated variants. **B** Venn diagram of ESKD-associated variants. The four pathogenicity classification categories include ClinVAR, CADD, Polyphen-2, and SIFT

Among the 459 CKD-associated variants, 280 were classified as pathogenic. Figure 2A illustrates the distribution of pathogenic variants across four categories. Notably, 10 variants fulfilled all pathogenicity criteria (Table 2). Additionally, 80 variants fulfilled three criteria, 85 fulfilled two, 106 fulfilled one, and 174 missense variants were not predicted to be pathogenic. On the other hand, among the 473 ESKD-associated variants, 279 were classified as pathogenic. Figure 2B illustrates the distribution of these variants across four classification categories, with 6 variants meeting all pathogenic criteria (Table 2). Additionally, 92 variants fulfilled three criteria, 90 fulfilled two, 91 fulfilled one, and 186 missense variants were not predicted to be pathogenic. We highlighted 14 variants predicted to be pathogenic by ClinVAR, CADD, Polyphen-2, and SIFT (Table 2). Notably, rs147047715 on *PDE6A* and rs201835496 on *PROKR2* have been observed to have a potential protective effect against both CKD and ESKD, as these variants were only present in healthy individuals but not in subjects with both conditions. In addition, the rs147047715 showed a protective effect on kidney function in an independent cohort of 1495 Taiwan Han Chinese from Taiwan Biobank (Supplementary Table 3).

**Table 2:** Pathogenic missense rare variants for CKD and ESKD

Chr.	Pos.	Ref.	Alt.	SNP id	Gene	Consequence	Amino acids	Allelic frequencies (EAS)			
								1KGP	gnomAD	TWB	
1	108929794	C	T	rs189033496	GPSM2	Missense	R/W	0.0030	0.0022	0.0030	
1	197103741	C	T	rs144969324	ASPM	Missense	G/D	0.0050	0.0066	0.0075	
4	158682269	G	A	rs121964954	ETFDH	Missense	A/T	0.0010	0.0017	0.0045	
5	149884570	T	C	rs147047715	PDE6A	Missense	I/V	0.0060	0.0033	0.0050	
7	92494621	G	T	rs144825021	PEX1	Missense	A/D	0.0069	0.0073	0.0055	
9	14842660	C	G	rs41298151	FREM1	Missense	G/A	0.0238	0.0181	0.0140	
10	101018879	C	T	rs201268590	PDZD7	Missense	A/T	0.0030	0.0041	0.0080	
11	62638996	G	C	rs200414966	GANAB	Missense	P/A	0.0040	0.0031	0.0025	
11	103170911	G	A	rs564701277	DYNC2H1	Missense	R/Q	0.0010	0.0061	0.0050	
14	91297376	G	A	rs142539336	CCDC88C	Missense	R/C	0.0030	0.0042	0.0015	
15	45103960	C	A	rs181461079	DUOX2	Missense	R/L	0.0040	0.0053	0.0120	
15	73367687	G	A	rs201375192	HCN4	Missense	A/V	0.0040	0.0049	0.0065	
16	57923327	G	C	rs201553871	CNGB1	Missense	P/R	0.0020	0.0061	0.0050	
20	5302662	C	G	rs201835496	PROKR2	Missense	W/S	0.0010	0.0029	0.0050	

Chr.	Pathogenicity				Carrier counts (N)			CKD		ESKD	
	ClinVAR	CADD	SIFT	Polyphen2	Normal	CKD	ESKD	P-value	OR	P-value	OR
1	Conflicting	31	Deleterious	Damaging	3	0	2	**3.63 × 10**^**–2**^	**0.0808**	3.38 × 10^–1^	0.3266
1	Conflicting	23.4	Deleterious	Damaging	5	1	4	**1.69 × 10**^**–2**^	**0.0955**	1.66 × 10^–1^	0.3878
4	Conflicting	28.8	Deleterious	Damaging	4	4	0	4.48 × 10^–1^	0.4898	**1.19 × 10**^**–2**^	**0.0600**
5	Conflicting	25.2	Deleterious	Possibly damaging	4	0	0	**1.19 × 10**^**–2**^	**0.0600**	**1.19 × 10**^**–2**^	**0.0600**
7	Conflicting	27.3	Deleterious	Damaging	3	0	2	**3.63 × 10**^**–2**^	**0.0808**	3.38 × 10^–1^	0.3266
9	Conflicting	21.7	Deleterious	Damaging	5	2	5	**4.33 × 10**^**–2**^	**0.1919**	3.10 × 10^–1^	0.4872
10	Conflicting	28.8	Deleterious	Damaging	4	2	0	9.79 × 10^–2^	0.2424	**1.19 × 10**^**–2**^	**0.0600**
11	Conflicting	22.3	Deleterious	Possibly damaging	3	1	0	1.10 × 10^–1^	0.1625	**3.63 × 10**^**–2**^	**0.0808**
11	Conflicting	31	Deleterious	Damaging	5	2	3	**4.33 × 10**^**–2**^	**0.1919**	1.22 × 10^–1^	0.2893
14	Conflicting	29.2	Deleterious	Damaging	4	1	2	**4.39 × 10**^**–2**^	**0.1206**	9.79 × 10^–2^	0.2424
15	Conflicting	35	Deleterious	Damaging	4	4	1	4.48 × 10^–1^	0.4898	**4.39 × 10**^**–2**^	**0.1206**
15	Conflicting	22.2	Deleterious	Damaging	3	0	2	**3.63 × 10**^**–2**^	**0.0808**	3.38 × 10^–1^	0.3266
16	Conflicting	23.3	Deleterious	Damaging	0	9	1	**3.19 × 10**^**–2**^	**9.4241**	1	1.0050
20	Conflicting	31	Deleterious	Damaging	3	0	0	**3.63 × 10**^**–2**^	**0.0808**	**3.63 × 10**^**–2**^	**0.0808**

Chr.: Chromosome; Pos.: Position; Ref.: Reference allele; Alt.: Alternative allele

### Replication of the reported genes from GWAS

Through the GWAS Catalog, we identified 2584 genes associated with kidney-related phenotypes, including 313 linked to CKD, 11 to ESKD, and 2260 to eGFR. The CKD cohort successfully replicated 50 of these genes, including 5 CKD-associated genes (SPI1, RIN3, *FTO*, *SIPA1L3*, and *EEF1E1*) and 45 eGFR-associated genes. Notably, several individual variants previously reported in GWAS for eGFR were also replicated, including rs3850625 in *CACNA1S* (CKD, p-value = 2.37 × 10^–2^, OR = 0.235), rs34823813 in *RNF123* (CKD, p-value = 2.43 × 10^–2^, OR = 7.452), and rs113956264 in *RPL3L* (CKD, p-value = 3.19 × 10^–2^, OR = 9.424). The ESKD cohort successfully replicated 48 of these genes, including 3 CKD-associated genes (*OTOGL*, *ADAMTS7*, and *LRIG1*) and 45 eGFR-associated genes.

### Replication of the established monogenic cause of kidney disease

Previous studies have identified several monogenic causal genes contributing to kidney disease development, including *APOL1*, *COL4A3*, *COL4A4*, *COL4A5*, *HNF1B*, *MUC1*, *PKD1*, *PKD2*, and *PKHD1*[7–9]. This study successfully observed monogenic effects within our cohort. Among CKD-associated genes, we replicated *PKD1* at rs550768338 (CKD, p = 3.63 × 10^–2^, OR = 0.081). Interestingly, this variant was carried by three healthy individuals but was absent in CKD subjects. For ESKD-associated genes, we replicated *COL4A4* at rs149117087 (ESKD, p = 4.33 × 10^–2^, OR = 0.192) and rs150979437 (ESKD, p = 4.09 × 10^–2^, OR = 4.261). rs149117087 was carried by five healthy individuals but found in only two ESKD cases, whereas rs150979437 was carried by two healthy individuals but present in 16 ESKD cases, indicating a stronger association with ESKD progression. While *MUC1*, a known causal gene for autosomal dominant tubulointerstitial kidney disease (ADTKD), was not detected in our study, we identified *MUC7*, *MUC3A*, and *MUC12*, which may suggest broader mucin-related pathways in kidney disease.

### Functional annotation of CKD and ESKD related genes

**Fig. 3:**
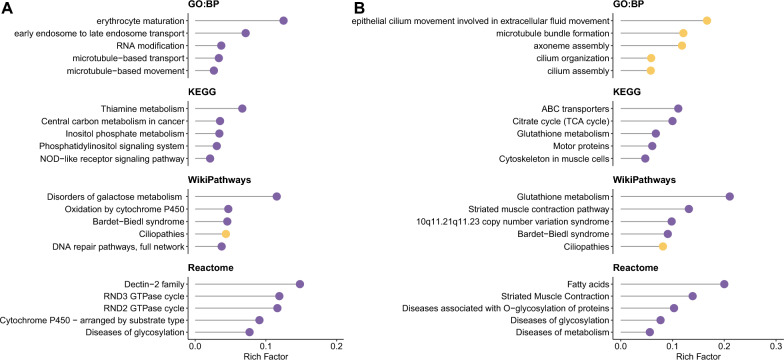
Pathway enrichment analysis of CKD- and ESKD-related genes. The top 5 enriched results from four databases were displayed: (**A**) GO: Biological Process, KEGG pathway, WikiPathways, and Reactome enrichment results for CKD; (**B**) GO: Biological Process, KEGG pathway, WikiPathways, and Reactome enrichment results for ESKD. Pathways with a false discovery rate (FDR) below 0.05 are highlighted in yellow, while others are shown in purple. The rich factor is the ratio of the number of input genes annotated in a pathway to the total number of genes annotated in the same pathway

To further explore the biological significance of CKD- and ESKD-associated genes, pathway enrichment analysis was performed separately for each condition. Figure 3 illustrates the enriched pathways for CKD (left panel) and ESKD (right panel), where each dot represents an enriched pathway. Notably, both CKD and ESKD share enrichment in ciliopathies and Bardet-Biedl syndrome, emphasizing the role of cilia dysfunction in kidney disease. In addition, ESKD-associated genes show strong enrichment in cilium assembly, cilium organization, cilium movement, and axoneme assembly, further reinforcing the role of ciliopathies in ESKD progression. Beyond ciliopathy-related pathways, CKD-associated genes are involved in NOD-like receptor signaling, phosphatidylinositol signaling, and inositol phosphate metabolism, suggesting inflammatory and metabolic dysregulation in CKD. Meanwhile, ESKD-associated genes exhibit enrichment in metabolic pathways, including glutathione metabolism, the citrate cycle (TCA cycle), and ABC transporters, indicating a strong link between oxidative stress, mitochondrial dysfunction, and ESKD. Additionally, diseases of glycosylation appear in both analyses, suggesting that defects in protein glycosylation may contribute to kidney disease progression in both CKD and ESKD.

### Correlation between polygenic score and kidney function

**Fig. 4:**
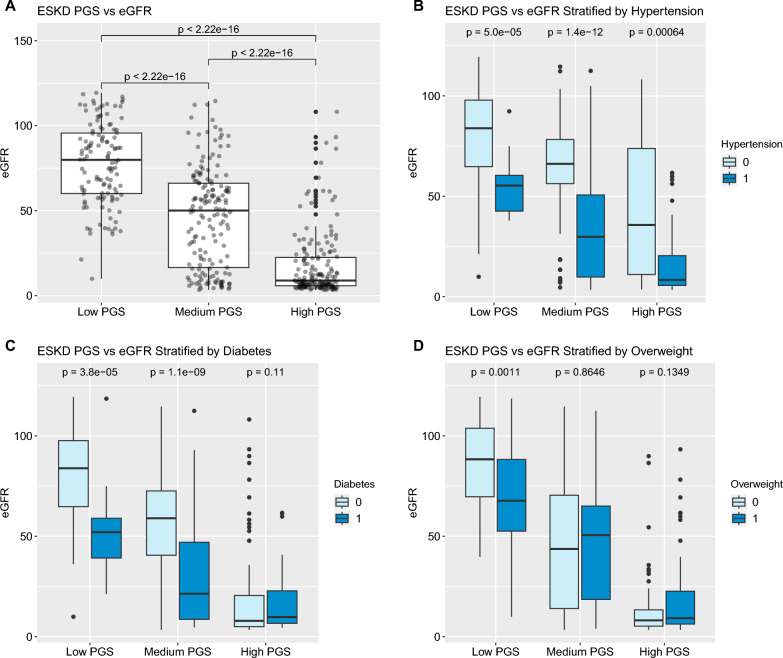
Association between ESKD polygenic scores and clinical characteristics. **A** Distribution of eGFR levels across ESKD PGS groups. **B** Comparison of eGFR levels among ESKD PGS groups stratified by hypertension status. **C** Comparison of eGFR levels among ESKD PGS groups stratified by diabetes status. **D** Comparison of eGFR levels among ESKD PGS groups stratified by overweight status

**Fig. 5:**
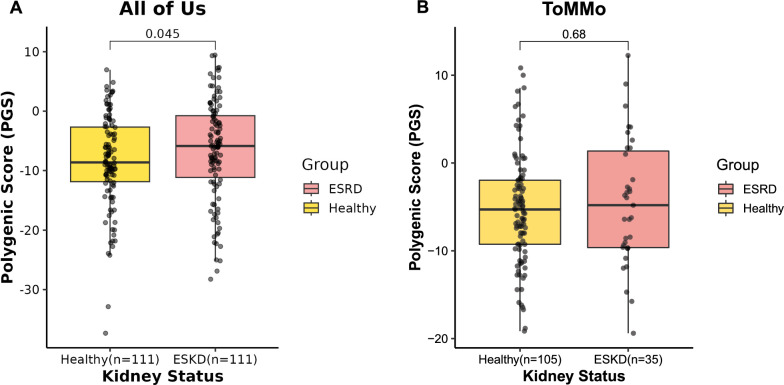
Validation of the ESKD PGS in the Asian population from the AoU and ToMMo. **A** Distribution of ESKD PGS between healthy individuals and ESKD subjects in the AoU Asian cohort. **B** Distribution of ESKD PGS between healthy individuals and ESKD subjects in the ToMMo cohort

To assess whether genetic risk variants could predict kidney function, we calculated PGS for CKD and ESKD based on the effect sizes of the identified candidate variants. A total of 459 and 473 variants were included in the calculations, respectively. We observed a Pearson correlation coefficient of − 0.13 (p = 0.0053) between the PGS for CKD and eGFR levels, and a substantially stronger correlation of − 0.71 (p < 2.2 × 10^–16^) between the PGS for ESKD and eGFR levels (Supplementary Fig. 1). These results indicate that the PGS for ESKD provides a much stronger prediction of kidney function. Based on this finding, we selected the PGS for ESKD as the primary score for subsequent analyses. We grouped individuals evenly by their PGS into low, medium, and high groups and compared eGFR levels across these groups. Subjects with an eGFR above 120 mL/min/1.73 m^2^ were excluded to remove outliers. After this exclusion, 122 subjects remained in the low PGS group, 165 in the medium group, and 166 in the high group (Fig. 4A). Our results indicate that the PGS for ESKD effectively predicts kidney function: individuals with lower PGS values had higher eGFR compared to those in the medium and high PGS groups (Fig. 4A). Significant differences in eGFR were observed among individuals with comorbidities, particularly hypertension and diabetes (Fig. 4B, C), with those affected showing lower eGFR levels. However, this trend was less pronounced in individuals with high PGS, suggesting that in those with a high genetic risk, kidney function is primarily influenced by genetics rather than the presence of diabetes. Additionally, overweight individuals (body mass index, BMI ≥ 23) in the low ESKD PGS group had lower eGFR levels (Fig. 4D). However, this trend was not observed in the medium and high PGS groups, whereas overweight individuals even showed slightly higher eGFR levels. These results suggest that a high genetic risk is a more dominant factor in determining kidney function compared to comorbidities such as diabetes and overweight status. Moreover, we included two independent cohorts to validate our findings. The first was from the Asian population of the AoU, comprising 111 ESKD individuals and 111 healthy controls. A significant difference in PGS was observed between the two groups (p-value = 0.045), with ESKD individuals exhibiting a higher PGS (Fig. 5A). For further validation, we utilized data from the ToMMo, where 35 ESKD cases and 105 healthy controls were included. In this cohort, the average PGS was higher in the ESKD group (mean = − 4.51) compared to the healthy group (mean = − 5.29). However, this difference was not statistically significant (p-value = 0.68, Fig. 5B).

## Discussion

Our study utilized WES to investigate the genetic basis of CKD and ESKD in a hospital-based cohort of 500 Taiwanese individuals. Participants were carefully selected through rigorous clinical evaluation to ensure precise phenotype classification. We successfully identified both monogenic and polygenic disease-associated genes, highlighting the crucial role of ciliary function, inflammation, and metabolic pathways in CKD and ESKD. Additionally, we constructed a genome-wide polygenic score for ESKD, which effectively predicted kidney function. Validation using the AoU further confirmed the PGS’s ability to predict ESKD susceptibility in Asian populations. Our study advances current knowledge by uncovering the impact of rare variants in CKD and ESKD, emphasizing the importance of genetic risk in clinical intervention and precision medicine.

Cameron-Christie et al. identified several CKD-associated genes from WES data, including *PKD1*, *PKD2*, *COL4A5*, *COL4A3*, and *COL4A4* [11]. Our study successfully replicated *PKD1* and *COL4A4*, reinforcing their significant role in CKD and ESKD. Germline pathogenic mutations in *PKD1* are known to cause autosomal dominant polycystic kidney disease (ADPKD) [19], a condition that increases the risk of developing ESKD over time. Interestingly, we identified a *PKD1* variant, rs550768338, in three healthy individuals and three ESKD subjects, but not in CKD subjects. The detection of *PKD1* variants in healthy individuals may represent incidental early genetic diagnosis in asymptomatic carriers, highlighting the value of genetic testing in nephrology. Additionally, we identified *COL4A4* variants rs149117087 and rs150979437. *COL4A4* is associated with both autosomal dominant and recessive Alport syndrome (ADAS and ARAS), a condition characterized by glomerulonephritis, ESKD, and hearing loss [20, 21]. While ClinVar classifies rs149117087 as benign for Alport syndrome, this variant was observed in 5 healthy individuals, 3 CKD patients, and 2 ESKD patients in our cohort. Functional studies are needed to confirm the biological role of *COL4A4* in CKD and ESKD. In contrast, rs150979437 was carried by 2 healthy individuals, 9 CKD patients, and 16 ESKD patients, potentially implicating a stronger genetic effect on kidney disease progression. Furthermore, Gbadegesin et al. demonstrated an association between *APOL1* G1 and G2 variants and increased CKD risk among West Africans [7]. However, *APOL1* variants were not detected in our study, likely due to population differences.

Our study also identified several kidney-associated genes previously identified in GWAS. This suggests that they may contribute to kidney disease through both common and rare variants, demonstrating the complex genetic architecture of CKD and ESKD. Notably, the MAF of rs3850625 in *CACNA1S* is relatively low in the Taiwanese population (MAF = 0.024 in our cohort) compared to Europeans (MAF = 0.1183 in 1 KG European data). A similar trend was observed for rs34823813 in *RNF123* (MAF = 0.023 in our cohort; 1 KG European = 0.1113) and rs113956264 in *RPL3L* (MAF = 0.016 in our cohort; 1 KG European = 0.0288). These findings indicate the potential differences in the pathogenicity of variants across populations, likely influenced by distinct inheritance patterns and ancestral genetic backgrounds. Among the novel genes with rare pathogenic variants (Table 2), *PEX1* was linked to Zellweger spectrum disorder, a genetic disorder that causes brain, liver, and kidney problems in newborns [22]. Variants in the *GANAB* gene have been implicated in autosomal dominant polycystic kidney disease, a condition characterized by the formation of renal cysts leading to progressive kidney dysfunction [23]. The *DYNC2H1* gene encodes a dynein motor protein essential for retrograde ciliary transport and is linked to ciliopathies such as short rib-polydactyly syndrome, which involves renal abnormalities [24]. Our findings align with previous studies emphasizing the role of ciliary dysfunction in kidney disease and further support its involvement in CKD and ESKD progression. Furthermore, *PROKR2* mutations are associated with Kallmann syndrome, a rare disease characterized by delayed puberty, impaired sense of smell, and kidney abnormalities, indicating a possible link to kidney development disorders [25].

Interestingly, the majority of identified variants exhibited a protective effect rather than increasing disease risk (Table 2). Specifically, the variant rs147047715 on *PDE6A* was found exclusively in four healthy individuals but was absent in CKD and ESKD cases. Similarly, rs201835496 on *PROKR2* was carried by three healthy individuals but was not detected in CKD or ESKD patients. While these observations are intriguing, they should be interpreted with caution given the limited sample size and the lack of functional validation. These findings support a hypothesis that kidney disease progression may be driven by both loss of protective mechanisms and the gain of risk factors. This perspective could shift future research and therapeutic strategies toward identifying and preserving such protective factors in kidney function. Future research should explore pharmacological interventions that stabilize or mimic the effects of these protective variants, potentially leading to new avenues in precision medicine for kidney disease management.

Pathway enrichment analysis highlighted a significant role for ciliopathy-related pathways in both CKD and ESKD. Disruptions in ciliary signaling pathways contribute to nephronophthisis, polycystic kidney disease, and other renal disorders [26]. The shared enrichment of ciliopathies and Bardet-Biedl syndrome genes in both CKD and ESKD suggests that ciliary dysfunction is a fundamental contributor to kidney disease susceptibility and progression [27]. Beyond ciliopathies, our study also revealed significant enrichment of inflammatory and metabolic pathways in CKD and ESKD. Chronic inflammation is a well-recognized factor in kidney disease, contributing to fibrosis, endothelial dysfunction, and disease progression [28]. Specifically, phosphatidylinositol signaling is crucial for kidney cell survival, autophagy, and inflammation. A key component of this pathway, PI3K/AKT signaling, regulates cellular responses to stress and has been shown to inhibit apoptosis, thereby mitigating kidney damage by downregulating the expression of NOD [29]. In addition, studies suggest that targeting NOD—like receptors could be a new strategy for the treatment of numerous kidney diseases [30].

The PGS analysis demonstrated that ESKD risk variants serve as potential predictors of kidney function, which could offer possible clinical utility for early risk stratification. Individuals with lower ESKD PGS exhibited significantly higher eGFR, reinforcing the crucial role of rare genetic risk factors in kidney function decline. Our findings indicate that individuals with a high genetic risk may benefit from early interventions such as routine renal function monitoring, stricter blood pressure control, and nephroprotective treatments (e.g., RAAS inhibitors, SGLT2 inhibitors) to mitigate disease progression. On the other hand, for individuals with lower genetic risks, comorbidities like diabetes and obesity play a more important role in determining kidney function. This highlights the importance of lifestyle modifications, including weight management, glycemic control, blood pressure control, and cardiovascular risk reduction, as primary strategies for kidney health preservation.

In this study, the predictive value of our PGS for ESKD was validated in the AoU Asian cohort, where a significant difference in PGS was observed between ESKD and healthy individuals. We further test our PGS model in the ToMMo cohort. Despite not reaching statistical significance, results in the ToMMo cohort exhibited a trend consistent with findings from both Taiwanese and AoU cohorts. This lack of significance is likely attributed to the relatively small number of ESKD cases in the ToMMo cohort (N = 35). These findings reveal the potential of the ESKD PGS as a precision medicine tool for kidney risk stratification, supporting the development of personalized intervention strategies based on an individual’s genetic risk profile. However, further studies are required to confirm the clinical utility of the PGS risk stratification for kidney disease management.

Our study has several limitations. First, the pathogenicity of identified variants was inferred from previous studies and in silico predictive algorithms, which can produce inconsistent results. For instance, ClinVar provides disease specific clinical significance based on reported cases and published literature, but conflicting interpretations between submitters may occur. In contrast, other in silico tools used in this study are not disease-specific and rely purely on predictive models. Without direct functional validation, these predictions should be interpreted with caution. Further experimental studies are needed to confirm the biological role of these variants in CKD and ESKD. Second, the relatively modest sample size may lead to false positive results. To address this, we evaluated the predictive ability of our PGS across the entire TMU cohort (N = 500), the AoU ESKD Asian cohort (N = 222), and the ToMMo ESKD cohort (N = 140). With the study cohorts of Asian populations, our study provides valuable genomic insights into an under-represented population in previous WES study [11]. However, future studies will be needed to validate our findings in larger and more diverse populations. Lastly, while rare variants often exhibit larger effect sizes, they do not account for the full genetic contribution to disease susceptibility. Future studies integrating both rare and common variants in a larger cohort with longitudinal follow-up data are needed for a more comprehensive understanding of CKD and ESKD genetics.

## Conclusion

Using a hospital-based cohort with strict clinical diagnostic criteria, our study ensures precise phenotype classification, enhancing the reliability of genetic discoveries. The unique genetic background of the Taiwanese population successfully replicated monogenic kidney disease genes and identified GWAS-reported genes, emphasizing the role of rare variants. Beyond these, we discovered rare variants in novel genes linked to ciliopathies, inflammation, and metabolic dysfunction, suggesting their potential involvement in kidney disease progression. Moreover, our findings show the potential utility of the PGS for ESKD as a predictor of kidney function and disease susceptibility. We suggest that individuals with high genetic risk may benefit from earlier interventions, whereas those with lower PGS may be effectively managed through lifestyle modifications targeting comorbidities associated with kidney diseases. Future studies in larger populations are needed to confirm the clinical utility of the PGS risk stratification for kidney disease management.

## Supplementary Information

Supplemental Material 1: Table 1. Missense Rare Variants for CKD.

Supplemental Material 2: Table 2. Missense Rare Variants for ESKD.

Supplemental Material 3: Table 3. Association analysis between pathogenic variants and eGFR in the Taiwan Biobank.

Supplemental Material 4: Figure 1. Correlation between eGFR levels and PGS for CKD and ESKD.

## Data Availability

The individual-level genomic and phenotype data is available upon request or application to the TMU biobank (https://ohr.tmu.edu.tw/en/about/34), the AoU research program (https://www.researchallofus.org/), and the ToMMo biobank (https://www.megabank.tohoku.ac.jp/english/sample/) for research purposes. The data supporting the findings of this study are available in main text or the supplementary materials.

## Abbreviations

CKD: Chronic kidney disease
ESKD: End-stage kidney disease
WES: Whole-exome sequencing
PGS: Polygenic score
eGFR: Estimated glomerular filtration rate
GWAS: Genome-wide association studies
TMU: Taipei Medical University
AoU: All of us research program
ToMMo: Tohoku medical megabank organization
uPCR: Urine protein-creatinine ratio
uACR: Urine microalbumin-creatinine ratio
IRB: Institutional review board
MAF: Minor allele frequency
