# Supplementary figures and images for: Whole exome sequencing and polygenic risk assessment for kidney functions and clinical management in both hospital-based cohort and population-based Asian cohorts
Source: J Biomed Sci. 2025 Aug 6;32:72. doi: 10.1186/s12929-025-01168-0 (PMC12330128; doi:10.1186/s12929-025-01168-0)

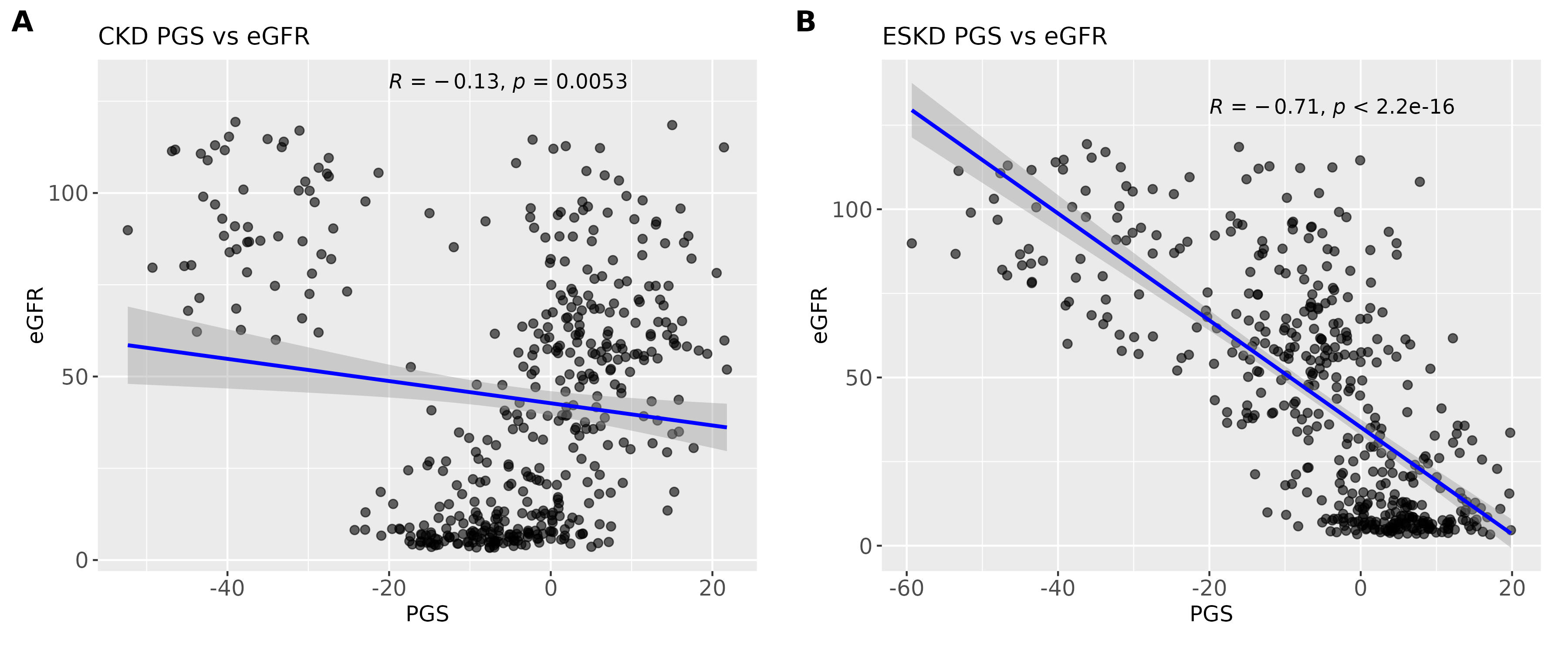

Supplement: Supplementary file 1 — Supplemental Material 1: Table 1. Missense Rare Variants for CKD. [file 12929_2025_1168_MOESM1_ESM.jpg]
